# Salivary lactoferrin is associated with cortical amyloid-beta load, cortical integrity, and memory in aging

**DOI:** 10.1186/s13195-021-00891-8

**Published:** 2021-09-06

**Authors:** Lucia Reseco, Mercedes Atienza, Marina Fernandez-Alvarez, Eva Carro, Jose L. Cantero

**Affiliations:** 1 Laboratory of Functional Neuroscience, Pablo de Olavide University, Ctra. de Utrera Km 1, 41013 Seville, Spain; 2 CIBERNED, Network Center for Biomedical Research in Neurodegenerative Diseases, Madrid, Spain; 3 Group of Neurodegenerative Diseases, Hospital 12 de Octubre Research Institute (imas12), Madrid, Spain

**Keywords:** Saliva, Lactoferrin, Aging, Amyloid-beta PET, FDG-PET, Cortical thickness

## Abstract

**Background:**

Aging is associated with declining protective immunity and persistent low-grade inflammatory responses, which significantly contribute to Alzheimer’s disease (AD) pathogenesis. Detecting aging-related cerebral vulnerability associated with deterioration of the immune system requires from non-invasive biomarkers able to detect failures in the brain-immunity connection. Reduced levels of salivary lactoferrin (sLF), an iron-binding protein with immunomodulatory activity, have been related to AD diagnosis. However, it remains unknown whether decreased sLF is associated with increased cortical amyloid-beta (Aβ) load and/or with loss of cortical integrity in normal aging.

**Methods:**

Seventy-four cognitively normal older adults (51 females) participated in the study. We applied multiple linear regression analyses to assess (i) whether sLF is associated with cortical Aβ load measured by 18F-Florbetaben (FBB)-positron emission tomography (PET), (ii) whether sLF-related variations in cortical thickness and cortical glucose metabolism depend on global Aβ burden, and (iii) whether such sLF-related cortical abnormalities moderate the relationship between sLF and cognition.

**Results:**

sLF was negatively associated with Aβ load in parieto-temporal regions. Moreover, sLF was related to thickening of the middle temporal cortex, increased FDG uptake in the posterior cingulate cortex, and poorer memory. These associations were stronger in individuals showing the highest Aβ burden.

**Conclusions:**

sLF levels are sensitive to variations in cortical Aβ load, structural and metabolic cortical abnormalities, and subclinical memory impairment in asymptomatic older adults. These findings provide support for the use of sLF as a non-invasive biomarker of cerebral vulnerability in the general aging population.

**Supplementary Information:**

The online version contains supplementary material available at 10.1186/s13195-021-00891-8.

## Background

Evidence suggests that aging modifies the composition of the oral microbiome favoring dysbiosis, increased infections, and persistent low-grade inflammation, which may ultimately compromise general health [1, 2]. Oral infections increase the morbidity and mortality risk in older individuals [3] and the susceptibility to develop systemic diseases, such as cardiovascular [4] and respiratory diseases [5], diabetes mellitus [6], rheumatoid arthritis [7], and dementia [8].

Lactoferrin (LF) is an iron-binding glycoprotein synthesized by neutrophil granulocytes and released by exocrine glands [9]. Among its multiple functions [10], LF contributes to host defense against infections by sequestering the ionic iron required for microbial growth [11–14]. In saliva, LF is modulated by the inflammatory state of the oral mucosa and plays a central role in regulating the oral microbiome [15].

LF has the capacity to penetrate into the brain parenchyma via receptor-mediated transcytosis [16–18]. Thus, exogenous administration of LF has been associated with improved spatial cognition through reductions in oxidative stress and inflammation in the hippocampus of aged mice [19], with reduced motor deficits in an induced mouse model of Parkinson disease (PD) [20, 21] and with decreased amyloid-beta (Aβ) aggregation and enhanced cognition in transgenic mouse models of Alzheimer’s disease (AD) [22, 23]. In humans, LF has been shown to accumulate in dopamine neurons of PD patients [24] and in cortical regions affected by AD pathology [25–27], likely to attenuate disease consequences. In line with this, LF administration in AD patients led to a reduction of AD pathology and improvement of cognitive performance [28].

Recent evidence suggests that salivary LF (sLF) could be an early, non-invasive, and cost-effective AD biomarker [29, 30]. In these studies, we showed that sLF levels are reduced in patients with mild cognitive impairment (MCI) and AD compared to healthy controls [29]. We next demonstrated that low concentrations of sLF were able to differentiate positive Aβ-MCI/AD patients not only from controls but also from patients with frontotemporal dementia [30]. However, it remains unknown whether sLF is related to cortical Aβ load in clinically normal older adults. This aspect deserves attention since Aβ depositions are ubiquitous in the brain of a high proportion of asymptomatic older individuals [31–33], and they are further considered an early pathological event in AD [34, 35]. To shed light into this, we evaluated associations between sLF and cortical Aβ burden measured with ^18^F-Florbetaben (FBB) amyloid positron emission tomography (PET) imaging. Our hypothesis is that reduced levels of sLF are related to increased Aβ load in clinically normal older adults, mainly in those cortical regions particularly vulnerable to accelerated aging and AD.

Regional patterns of cortical thinning and cortical hypometabolism are well-established signatures of prodromal and clinical AD [36–39]. However, patterns of cortical thickening and cortical hypermetabolism have also been associated with increased cerebral Aβ aggregates in asymptomatic older adults [40–43], likely signaling cerebral vulnerability and/or neural compensation in response to increased Aβ deposition. Based on these findings, we expect that sLF levels are negatively correlated with cortical thickness and cortical glucose metabolism in normal older individuals and that such relationships are stronger in those participants showing higher Aβ load. As previous studies have found associations between LF and improved memory [19, 44], we further predict that sLF-related variations in cortical thickness and/or FDG/FBB binding are associated with subclinical changes in memory function compared to other cognitive domains.

## Methods

### Subjects

Seventy-four cognitively normal older adults participated in the study (age = 66.5 ± 5.7 years; 51 females). They were recruited from senior citizen’s associations, health-screening programs, and hospital outpatient services. All of them underwent neurological and neuropsychological assessment to discard the presence of early signs of dementia and objective cognitive impairment. Participants met the following inclusion criteria: (i) normal global cognitive status in the MMSE (scores ≥ 26), (ii) normal cognitive performance in the neuropsychological tests relative to appropriate reference values for age and education level, (iii) global score of 0 (no dementia) in the Clinical Dementia Rating (CDR), (iv) functional independence as assessed by the Spanish version of the Interview for Deterioration in Daily Living Activities [45], (v) scores ≤ 5 (no depression) in the short form of the Geriatric Depression Scale [46], and (vi) not be on medications that affect cognition. All participants gave informed consent to the experimental protocol approved by the Ethical Committee for Clinical Research of the Junta de Andalucía according to the principles outlined in the Declaration of Helsinki. Table 1 shows sample characteristics.

**Table 1:** Demographics and cognitive and cerebral Aβ measures

	Total sample *N* = 74	Female *N* = 51	Male *N* = 23
Age	66.5 ± 5.7	66.0 ± 5.9	67.6 ± 5.1
Education years	12.5 ± 4.9	12.5 ± 5.2	12.4 ± 4.1
ApoE4 (yes/no)	16/58	11/40	6/17
CDR	0	0	0
MMSE	29.0 ± 1.2	28.9 ± 1.2	29.3 ± 1.1
Memory Binding Test			
Total free recall	15.5 ± 4.4	15.8 ± 4.3	14.9 ± 4.7
Pairs in free recall	5.9 ± 2.8	6.0 ± 2.7	5.8 ± 3.0
Total paired recall	24.6 ± 4.2	24.6 ± 3.9	24.7 ± 4.8
Paired recall pairs	9.7 ± 3.7	9.7 ± 3.8	9.7 ± 3.5
Total delayed free recall	16.4 ± 4.9	16.6 ± 4.8	16.0 ± 5.1
Pairs in delayed free recall	6.3 ± 3.1	6.4 ± 3.0	6.1 ± 3.3
Total delayed paired recall	24.0 ± 4.6	23.9 ± 4.4	24.1 ± 5.2
Letter-Number Test	9.0 ± 2.3	8.8 ± 2.1	9.7 ± 2.7
Digit span	14.3 ± 3.2	14.1 ± 3.0	14.9 ± 3.4
D2 Test of Attention	364.1 ± 94.0	355.6 ± 107.0	382.8 ± 52.8
TMT-A	47.0 ± 21.5	47.6 ± 21.5	45.6 ± 22.0
TMT-B	119.5 ± 67.7	123.4 ± 69.6	110.8 ± 63.8
Tower of London	319.3 ± 113.2	338.6 ± 121.4	276.3 ± 78.8
Boston Naming Test	12.1 ± 2.1	11.9 ± 2.1	12.5 ± 1.9
Salivary LF (μg/ml)	6.2 ± 2.9	6.0 ± 2.9	6.5 ± 2.8
Saliva total protein (μg/ml)	4.1 ± 1	4.1 ± 1.1	4.2 ± 1
Global Aβ load (SUVR)	1.2 ± 0.3	1.1 ± 0.3	1.2 ± 0.3
FBB-PET (positive Aβ)	10	7	3

Results are expressed as mean ± SD, unless otherwise stated. *CDR* Clinical Dementia Rating, *MMSE* Mini Mental State Examination, *TMT-A and B* Trail Making test (forms A and B), *LF* lactoferrin, *SUVR* standardized uptake value ratios, *FBB* Florbetaben

### Neuropsychological assessment

The neuropsychological battery included different tests for assessment of memory, working memory, attention, executive function, and language. We computed a composite *Z* score for each cognitive domain comprising the following tests: (i) the Spanish version of the Memory Binding Test as an index of memory [47], (ii) the letter-number sequencing (Wechsler Adult Intelligence Scale-III) and the digit span (Wechsler Memory Scale-III) subtests as measures of working memory, (iii) the D2 test as an index of attention, (iv) the Trail Making test (TMT, forms A and B) and the Tower of London as measures of executive function, and (iv) the short form of the Boston Naming Test as a measure of language.

### Quantification of sLF

Saliva samples were collected at 9:00–10:00 am in all subjects using the spitting method. Participants were asked to refrain from any oral stimulation (e.g., food, drink, smoke, chew gum, oral hygiene) or taking medication for at least 8 h prior to saliva collection. They were not allowed to wear lipstick or lip balm. The presence of blood contamination in saliva samples was excluded by visual inspection. Unstimulated whole saliva was collected into 50 ml conical sterile polypropylene tubes previously treated with 2% sodium azide solution. Samples were immediately placed on ice and centrifuged at 365*g* for 5 min at 4 °C. Supernatant saliva was then transferred into 1.5 ml polypropylene tubes, treated with a protease inhibitor cocktail (cOmplete Ultra Tablets mini, Roche, Basel, Switzerland), and stored at − 80 °C until analysis. Estimation of total protein concentration in saliva samples was performed with a bicinchoninic acid assay (BCA) (Pierce, Rockford, IL), according to the manufacturer’s instructions. We further correlated sLF with saliva protein levels to determine if salivary flow rate was associated with sLF concentration [48].

**Fig. 1:**
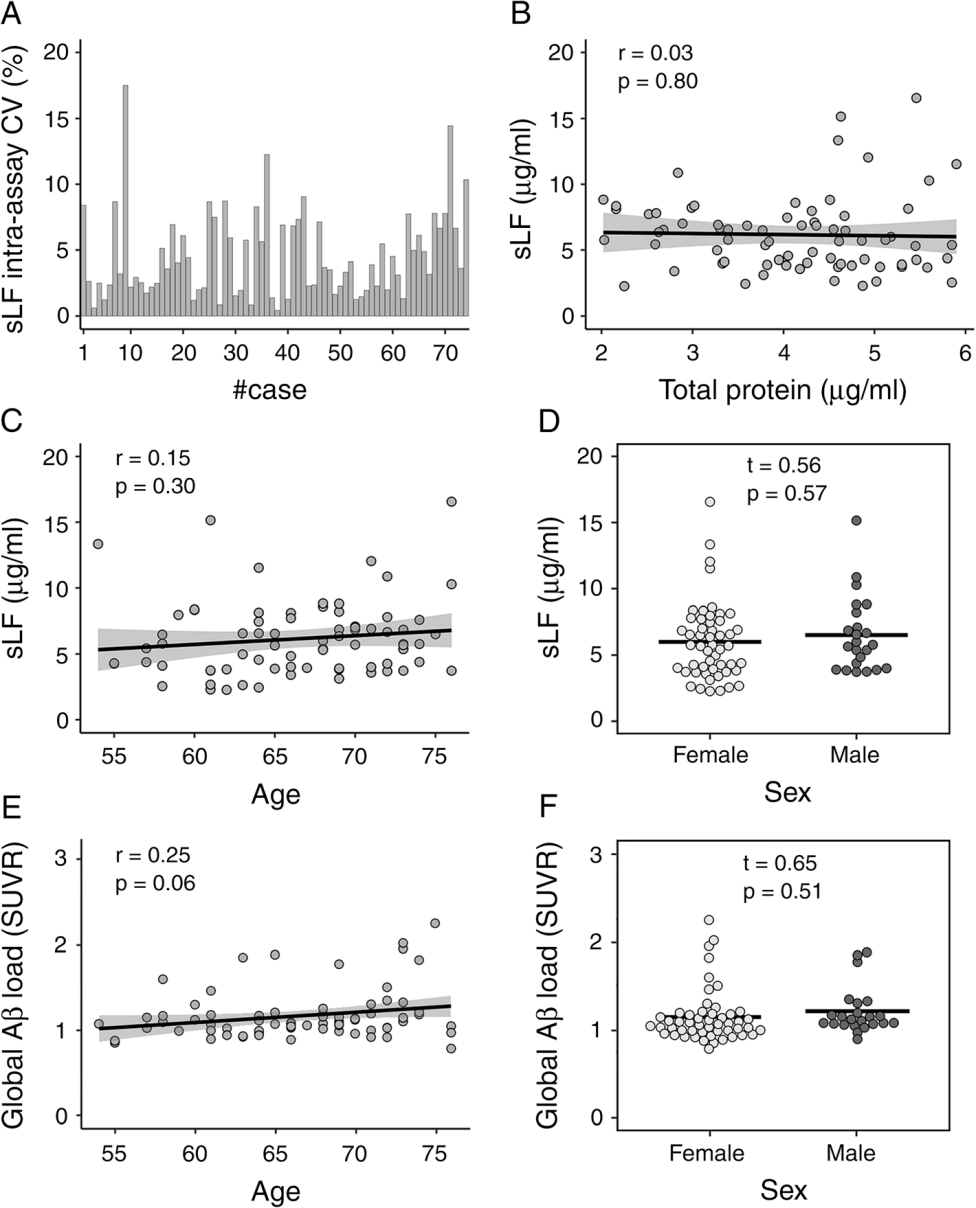
Effect of age and sex on sLF levels and global Aβ load. **A** Intra-assay coefficient of variation (CV) of sLF for each participant. **B** Relationship between salivary total protein content and sLF. **C** Relationship between age and sLF adjusted by sex. **D** sLF differences between sexes adjusted by age. **E** Association of age with global Aβ load (SUVR) adjusted by sex. **F** Sex differences in cortical Aβ load adjusted by age

Levels of sLF were quantified using a commercially available ELISA kit (ab200015, Abcam, Cambridge, UK) according to manufacturer’s instructions. Briefly, we added 50 μL of saliva samples or standard to appropriate wells diluted 1:10,000 into sample diluent NS. All samples were tested in duplicate and the average of the two measurements (μg/ml) was used for statistical analyses. Mean intra- and inter-assay coefficients of variation (CV) were 4.6% and 8%, respectively. Intra-assay CV values were below 20% in all subjects (Fig. 1A).

### MRI and PET acquisition

Structural brain images were acquired on a Philips Ingenia 3 T MRI scanner equipped with a 32-channel head coil (Philips, Best, Netherlands). A whole-brain T1-weighted magnetization prepared rapid gradient echo (MPRAGE) sequence was acquired in the sagittal plane using the following parameters: repetition time (TR) = 2600 ms, echo time (TE) = 4.7 ms, flip angle = 9°, matrix = 384 × 384, voxel resolution = 0.65 mm^3^ isotropic, and no gap between slices. Head motion was minimized by using a head restraint system and placing foam padding around the subject’s head. Participants were provided with headphones and foam earplugs to attenuate scanner noise.

PET examinations (2-[^18^F]fluoro-2-deoxy-D-glucose—FDG—and ^18^F-Florbetaben—FBB) were performed in a Philips Gemini 16 PET/CT scanner (Philips, Best, Netherlands). FDG-PET assessments were followed by FBB-PET scans two weeks later. Subjects fasted for at least 8 h before FDG-PET examination. Participants were injected with 185 MBq of FDG in a quiet, dimly lit room. FDG-PET images were acquired 30 min after injection with scan duration of 10 min. For FBB-PET imaging, participants were injected with 300 MBq of [^18^F] FBB (NeuraCeq™, Piramal Pharma) 90 min before acquisition. PET data were corrected for radioactive decay, dead time, attenuation, and scatter. Cerebral PET images were reconstructed iteratively with an isotropic voxel resolution of 2 mm^3^. Participants underwent a 20-min FBB-PET scan in dynamic mode consisting of 4 frames of 5 min each. Each frame was inspected for excessive motion. As no excessive head motion was detected in scanned images, the four frames were averaged to create a single static FBB brain image used for quantitative analysis. All FBB-PET scans were assessed visually by an expert in nuclear medicine and quantitatively using a standardized uptake value ratio (SUVR) cutoff of 1.43 [49].

### Estimation of cortical thickness, FDG, and FBB binding

MRI data were processed using the analysis pipeline of Freesurfer v6.0 (https://surfer.nmr.mgh.harvard.edu/) that involves intensity normalization, registration to Talairach, skull stripping, white matter (WM) segmentation, tessellation of WM boundaries, and automatic correction of topological defects [50]. Pial surface misplacements and erroneous WM segmentation were manually corrected on a slice-by-slice basis to enhance the reliability of cortical thickness measurements. Individual cortical thickness maps were smoothed using non-linear spherical wavelet-based de-noising schemes, which have previously shown greater specificity and sensitivity than Gaussian spatial filters for detecting local and global changes in cortical thickness [51]. Partial volume correction (PVC) of FDG/FBB PET images was performed with PetSurfer, a toolbox for PET analysis implemented in Freesurfer v6.0. We employed the Geometric Transfer Matrix-derived region-based voxel-wise method [52], assuming a uniform 6 mm point spread function. Cerebral FDG/FBB images were first co-registered to T1 scans. Next, PVC-cortical FDG/FBB images were transformed into SUVR using the pons or the grey matter of cerebellum as reference regions, respectively. Resulting PVC-FDG/FBB cortical-to-pons/cerebellum SUVR images were mapped into individual cortical surfaces and smoothed with non-linear spherical wavelet-based de-noising schemes [51]. Additionally, we obtained global cortical Aβ values for each participant using a FBB composite comprising 4 large bilateral cortical regions: frontal (orbitofrontal cortex/inferior frontal gyrus/middle frontal gyrus/superior frontal gyrus/frontal pole), cingulate (anterior/posterior/isthmus cingulate), parietal (precuneus/inferior parietal cortex/superior parietal cortex/supramarginal gyrus), and lateral temporal cortex (middle temporal/superior temporal gyri) [53].

### Statistical analysis

Simple linear regression was used to estimate the relationship of sLF with total protein content in saliva samples, while multiple linear regression was used to determine the association of sLF and global Aβ load with age and sex. We also applied multiple-linear regression analysis to test the hypothesis that sLF levels are associated with between-individual variations in cortical thickness and cortical FDG/FBB binding in clinically normal older adults. Cerebral dependent variables were Box-Cox transformed to improve normality and alleviate heteroscedasticity [54]. All statistical models, except the one performed with FBB binding, were adjusted for global Aβ load, age, and sex. The model that included FBB binding as dependent variable was only adjusted by age and sex. To determine whether the association of sLF with either cortical thickness or FDG uptake was moderated by global Aβ load, we added the sLF × global Aβ load interaction as explanatory variable. The two interaction models were adjusted by age, sex, sLF, and global Aβ load.

Vertex-wise regression analyses with cortical thickness and FDG/FBB binding as dependent variables were performed using the SurfStat package (https://www.math.mcgill.ca/keith/surfstat/). Results were corrected for multiple comparisons using a previously validated hierarchical statistical model [55]. This procedure first controls the family-wise error rate in significant clusters over smoothed statistical maps applying random field theory; and it next controls the false discovery rate in vertices of significant clusters over unsmoothed statistical maps. The anatomical location of clusters that survived correction was identified by the location of each cluster’s peak vertex on the Desikan-Killiany atlas [56]. For each regression analysis, we reported the maximum F (i.e., Roy’s largest root) and its associated *p* value, which was obtained by comparing either the additive or interactive model with a model with no regressors (i.e., a model that only included the intercept). For each post-hoc contrast, we reported the maximum *R*^2^ and its associated *p* value for each significant cluster. After inferential evidence of a main effect, we computed the standardized measure of effect size (i.e., Cohen’s *f*^2^), which allows an evaluation of local effect size within the context of a multivariate regression model [57]. To establish the precision of effect sizes, we computed 95% confidence intervals (*CI*_95%_) using the normal approximated interval with bootstrapped bias and standard error (*N* = 10.000 bootstrap samples) through the function *bootci* implemented in Matlab.

We next assessed associations between levels of sLF and cognition. Five cognitive domains were assessed: memory, working memory, attention, language, and executive function. To improve normality and heteroscedasticity, the model included as dependent variable the Yeo-Johnson transformation of composite *Z* scores for each cognitive domain [58]. We applied linear mixed modeling as this approach allows for random intercepts across participants, which, in turn, reduces de variance of fixed effect estimates [59]. In particular, we specified a four-step mixed effects model. The main predictors were added in the first step (i.e., cognitive domain, sLF, sLF-related variations in cortical Aβ load, cortical thickness and cortical FDG uptake, age, and sex), whereas the two-, three-, and four-way interactions were sequentially added in subsequent steps. We next applied ANOVAs to compare the different models. These analyses were performed with R Statistical Software v3.0.1 (R Foundation for Statistical Computing, Vienna, Austria).

### Power analysis

Power analysis was performed with the G*Power software (v3.1.9.6) (https://www.psychologie.hhu.de/arbeitsgruppen/allgemeine-psychologie-und-arbeitspsychologie/gpower.html). We computed an a priori (prospective) *power analysis* (fixed model, R^2^ deviation from zero) to achieve a satisfactory power level of 80% given a α-level of 0.05 and a Cohen’s effect size (*f*) of 0.25. As sex was included as covariate of no interest, the number of predictors ranged between 6 and 10. Taking into account these parameters, a sample size between 62 and 74 participants was required to achieve the desired power.

## Results

### Demographics, cognitive and cerebral Aβ measures

Table 1 includes demographics, cognitive and cerebral Aβ measures of the total sample and stratified by sex. The sample comprised 74 cognitively normal elderly subjects (51 females and 23 males). Ten of them showed a positive FBB scan (7 females and 3 males). sLF was not correlated with saliva protein levels (*r* = − 0.03, *p* = 0.8), arguing against an effect of salivary flow rate on sLF levels in our study (Fig. 1B). Regression analyses indicated that there was no main effect of age or sex on sLF (Fig. 1C, D) or global Aβ load (Fig. 1E, F). The age × sex interaction was also not significant for any of these dependent variables. On the contrary, we found a significant main effect of age on cognition, which was mostly evident for the memory domain, but found no significant differences between sexes or between cognitive domains (see Supplementary material).

### Relationship between sLF and cortical Aβ load

**Fig. 2:**
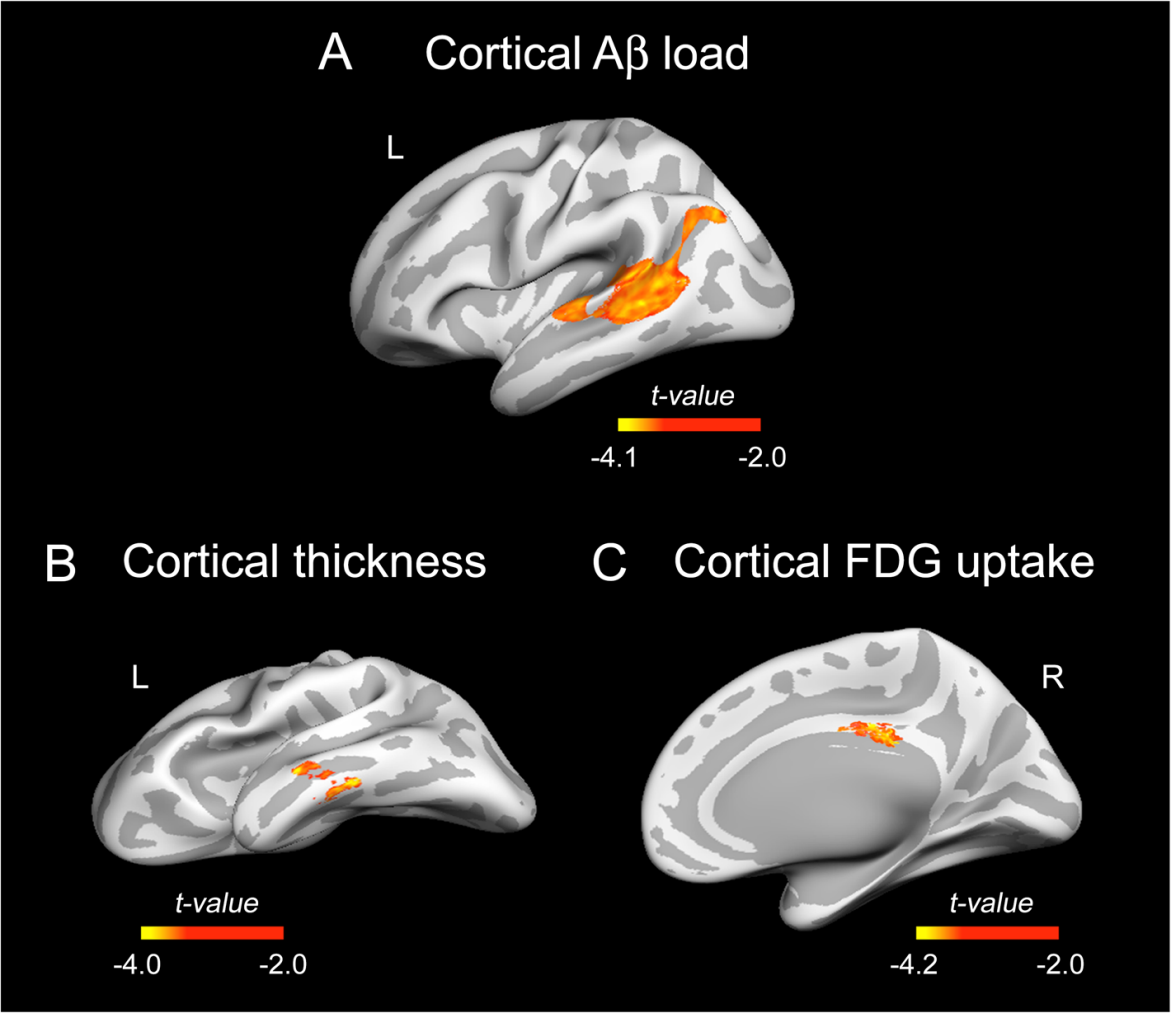
Association of sLF with cortical Aβ load, cortical thickness and cortical FDG uptake. The *t* statistics projected into the inflated cortical surfaces indicate the negative associations of sLF with cortical Aβ load (**A**) after adjustment for age and sex and with cortical thickness (**B**) and cortical FDG uptake (**C**) after adjustment for age, sex, and global Aβ load. All cortical measurements were Box-Cox transformed before whole-cortex vertex-wise analysis. Color bar indicates the range of significant *t* values. Left (L) and right (R)

Regression analysis revealed a main effect of sLF on cortical FBB binding after adjustment by age and sex (*F*_3,70_ = 8.3, *p* < 10^−4^). The post-hoc regression analysis showed a negative relationship between sLF levels and FBB binding in areas of the left temporal and inferior parietal lobe (${R}_{\mathrm{max}}^2$ = 0.22, *t*_max_ = 4.50, *p*_cluster_ = 0.006; *f*^2^ = 0.28, *CI*_95%_[0.21 0.35]). Results derived from this analysis are shown in Fig. 2A.

### Relationship between sLF and cortical thickness/cortical FDG uptake

After adjustment by global Aβ load, age, and sex, we also found a main effect of sLF on cortical thickness (*F*_4,69_ = 11,52, *p* < 10^−5^) and FDG uptake (*F*_4,69_ = 11.2, *p* < 10^−4^). Post hoc regression analyses showed a negative association of sLF with cortical thickness in areas of the left middle temporal lobe (${R}_{\mathrm{max}}^2$ = 0.19, *t*_max_ = 4.15, *p*_cluster_ = 0.008; *f*^2^ = 0.32, *CI*_95%_[0.25 0.38]) and with FDG uptake in areas of the right posterior cingulate cortex (rPCC) (${R}_{\mathrm{max}}^2$ = 0.2, *t*_max_ = 4.19, *p*_cluster_ = 10^−5^; *f*^2^ = 0.55, *CI*_95%_[0.48 0.62]). Results from these analyses are illustrated in Fig. 2B, C.

### Moderating role of global Aβ load on the relationship between sLF and cortical thickness/cortical FDG uptake

**Fig. 3:**
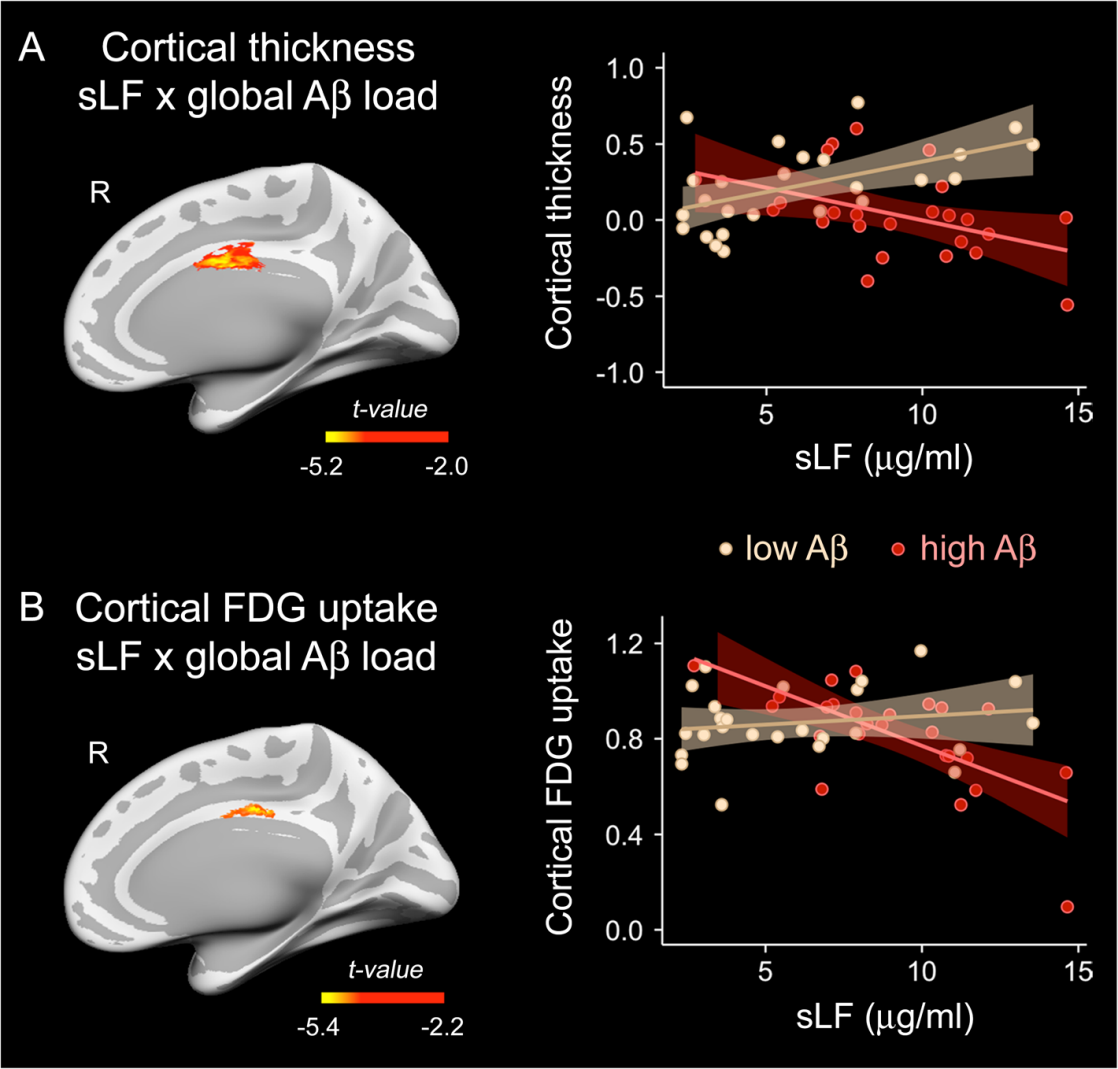
Moderating role of global Aβ load on the association of sLF with cortical thickness or cortical FDG uptake. Whole-cortex vertex-wise assessment of the two-way sLF × global Aβ load interaction on either cortical thickness (**A**) or cortical FDG uptake (**B**). The left panel shows the *t* statistics projected into the inflated cortical surfaces. The right panel shows the scatter plots for the relationship between sLF and the mean of the Box-Cox transformed values of thickness or FDG uptake within the right posterior cingulate cortex in subjects in the lowest and highest tertile of global Aβ load adjusted by age, sex, sLF, and global Aβ load. Color bar indicates the range of significant *t* values. Right (R)

To assess the putative role of global Aβ load as a moderator of the relationship between sLF and cortical thickness, we built a new model including the sLF × global Aβ load interaction term. The ANOVA comparing the additive and interaction models showed significant differences over the rPCC (*F*_5,68_ = 9.35, *p* < 10^−5^, ${R}_{\mathrm{max}}^2$ = 0.32, *t*_max_ = 5.76, *p*_cluster_ < 10^−6^; *rho* = 6.96, *CI*_95%_[3.8 10.1]). Figure 3A shows the results derived from this analysis on cortical surfaces and the scatter plot of the interaction effect. In particular, the correlation between sLF and cortical thickness was most negative for those individuals in the highest tertile of global Aβ load.

The interaction effect was also significant for the FDG uptake at the rPCC (*F*_5,68_ = 12.45, *p* < 10^−4^, ${R}_{\mathrm{max}}^2$ = 0.29, *t*_max_ = 5.39, *p*_cluster_ = 0.002; rho = 6.65, *CI*_95%_[4.4 8.9]). As illustrated in Fig. 3B, individuals in the highest tertile of global Aβ load showed a more negative relationship between sLF and cortical FDG uptake compared to those in the lowest tertile.

### Moderating role of sLF-related cortical variations on the association of sLF with cognition

We first built a mixed-effect model with cognition as dependent variable, with cognitive domain, sLF, age, sex, and sLF-related changes in Aβ load (i.e., the mean value of the significant Aβ cluster in Fig. 2A), cortical thickness (i.e., the mean value of the significant thickness cluster in Fig. 2B) and FDG uptake (i.e., the mean value of the significant FDG cluster in Fig. 2C) as fixed effects, and with participants as random effect. Results indicated that cognition was negatively associated with sLF (*F*_1,74_ = 10.4, *p* = 0.002), sLF-related changes in Aβ load (*F*_1,74_ = 4.3, *p* = 0.04), and age (*F*_1,74_ = 15.8, *p* = 0.0002).

**Fig. 4:**
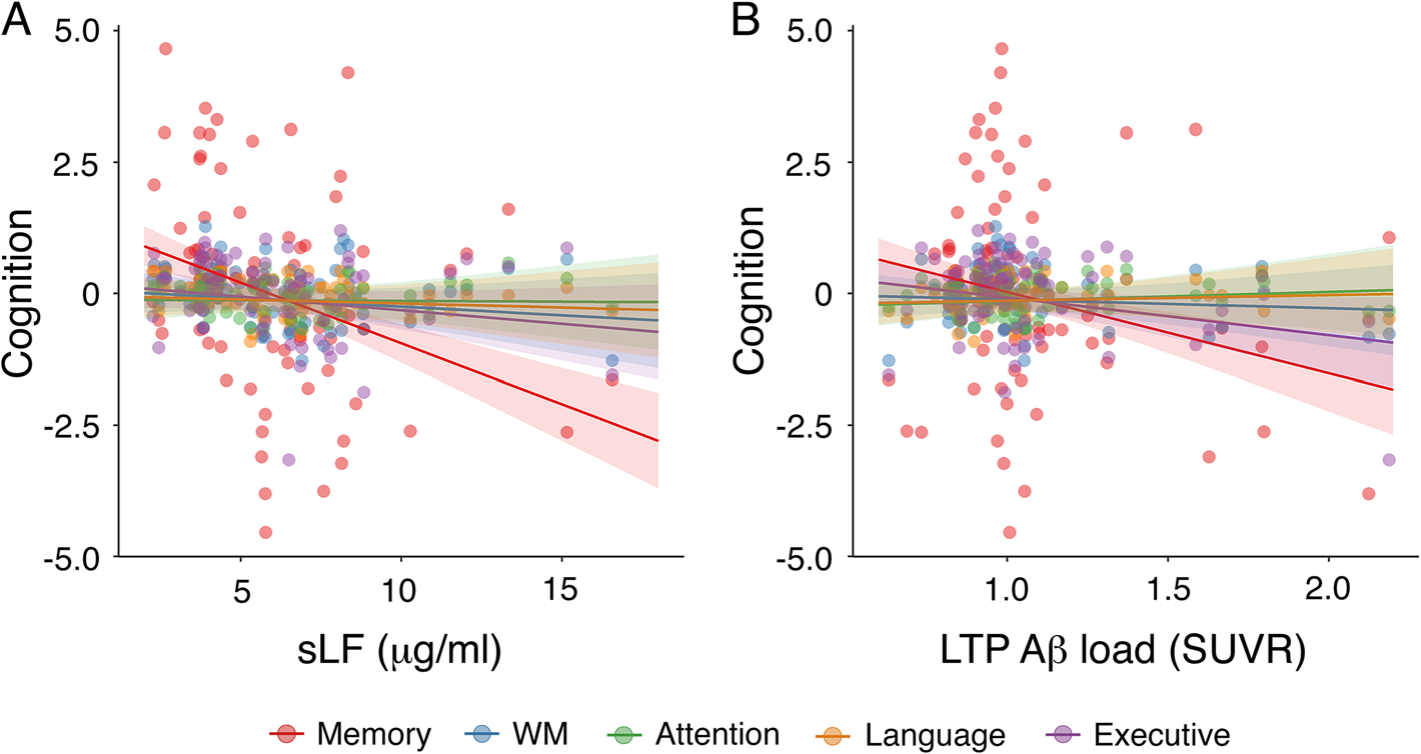
Association of sLF and sLF-related changes in cortical Aβ load with scores in different cognitive domains. **A** Relationship of sLF with cognition as a function of the cognitive domain. **B** Association of sLF-related changes in Aβ load over left temporo-parietal (LTP) regions (see Fig. 2A) as a function of the cognitive domain. WM, working memory. SUVR, standardized uptake value ratio

Next, we built an interaction model with 4 two-way interactions terms (i.e., cognitive domain × sLF, sLF-related changes in Aβ load, cortical thickness and FDG uptake). This model provided a better fit to the data than the additive model (*χ*^2^ = 38, *p* = 0.001). The ANOVA revealed a significant association of cognitive domain with sLF (*F*_4,296_ = 7, *p* = 0.00002) and with sLF-related changes in Aβ load (*F*_4,296_ = 4.2, *p* = 0.002). Post hoc analyses indicated that both sLF and sLF-related changes in Aβ load were negatively associated with memory when compared with the remaining cognitive domains. These results are illustrated in Fig. 4A and Fig. 4B, respectively.

We finally assessed whether these interactions were further moderated by sLF-related changes in either cortical thickness or cortical FDG uptake. But none of these models provided a better fit to the data when compared to the additive model or any of the interaction models mentioned above.

## Discussion

In this study, we showed that aging-related sLF is negatively associated with regional Aβ load, particularly in parieto-temporal regions of the left hemisphere. Results further revealed that global Aβ load moderated the association of sLF with increased thickness and FDG uptake in the rPCC. Remarkably, both sLF and sLF-related changes in Aβ load were associated with poorer memory. Together, these findings provide evidence supporting the role of sLF as a biomarker of cerebral vulnerability in normal aging and contribute to extend our understanding of sLF in the continuum aging-AD.

In consonance with previous results obtained in MCI and AD patients [29, 30], decreased levels of sLF were related to increased Aβ burden in left parieto-temporal regions of cognitively normal older adults. We have recently hypothesized that reduced sLF observed in prodromal and clinical stages of AD may indicate early immunological disturbances that eventually increase the risk of AD [60]. Low levels of sLF may assist oral dysbiosis, which, in turn, may produce long-term infections and a pro-inflammatory response that weakens the blood brain barrier, facilitating colonization of brain tissue by periodontal bacteria [61] and accelerating neuroinflammation that contribute to AD pathology [62]. In this vein, previous studies have shown an association between periodontal disease and brain Aβ accumulation in normal aging, suggesting that periodontal inflammation/infection may accelerate brain Aβ deposition [63, 64]. Our results complement these findings, suggesting that reductions of sLF relate to increased regional Aβ burden, which may reflect early immunological alterations potentially associated with higher risk of developing AD [60]. Alternatively, a recent study has shown that levels of LF in either saliva or cerebrospinal fluid (CSF) were not able to differentiate between controls, MCI/AD, and non-AD dementias. Nor did they find an association between sLF and CSF AD biomarkers [65]. These conflicting results do not invalidate our results that were obtained in cognitively normal elderly subjects, emphasizing the potential role of sLF to track cerebral Aβ changes in late life.

The present study further showed that between individual reductions in sLF are associated with thickening of the left middle temporal lobe and increased FDG uptake in the rPCC. Accumulated evidence suggests that cortical thickening of middle temporal regions occurs in cognitively normal elderly subjects before the onset of AD symptoms [42, 43, 66–68]. Accordingly, post mortem human brain studies have shown hypertrophy of neuronal cell bodies, nuclei, and nucleoli in asymptomatic individuals with Aβ aggregates, likely revealing a very early reaction to cerebral Aβ burden and/or the activation of cellular processes in an attempt to prevent the natural progression of AD [69]. Previous works have further identified a hypermetabolic phase in cognitively normal older adults, affecting the posterior cingulate among other cortical regions, which became hypometabolic in later stages of AD [70]. Patterns of increased cortical FDG uptake have also been observed in association with increased accumulation of Aβ deposition in asymptomatic older adults [40] that may result from Aβ-related microgliosis [71], overproduction of inflammatory mediators [72], and/or aberrant hyperactivation of cortical neurons instigated by Aβ burden [73]. Therefore, the link between decreased sLF and abnormal patterns of cortical thickness/cortical FGD uptake may mirror the earliest Aβ-related effects on vulnerable cortical regions in late life.

Interestingly, associations between decreased sLF and cortical thickening/cortical hypermetabolism were mostly evident in those individuals showing the highest global Aβ load. While it is increasingly clear that accumulation of Aβ plaques is not sufficient to cause AD [74], the mere presence of Aβ aggregates has adverse effects that might increase the risk for AD such as impaired microvascular integrity, unbalanced glucose homeostasis, failure in neuronal cell cycle control, and inflammatory responses regulated by microglia and astrocytes [75]. The moderating role of global Aβ burden on the relationship between sLF and cortical thickening/cortical hypermetabolism may reveal aggravated aging-related immunity deficits in individuals with the highest global Aβ concentration. Whether this association may eventually increase the likelihood of developing AD has to be determined in future studies. In the context of aging, substantial cerebral Aβ load could lead to chronic neuroinflammation favoring deregulation of the immune system and, consequently, altering the intrinsic immunomodulatory role of LF [76].

In line with this hypothesis, sLF-related increases in parieto-temporal Aβ load predicted lower cognition, particularly affecting memory. However, this relationship was not moderated by sLF-associated changes in either cortical thickness or FDG uptake. Previous studies have found greater LF deposition in brain regions enriched with Aβ plaques [25, 27], which may be interpreted as an attempt to minimize the consequences of cerebral amyloidosis. On the other hand, exogenous administration of LF has shown to stimulate the non-amyloidogenic processing of amyloid precursor protein and α-secretase activity, with a consequent reduction in Aβ deposition that ameliorates cognitive decline in mouse models of AD [22]. Beneficial effects of LF for the maintenance of cellular and tissue homeostasis may be due to an increase of autophagy activity via AMPK signaling through the LRP1 receptor [77].

## Limitations

The results of the current study are purely correlational, and we cannot infer any causal relationship between sLF, cerebral changes, and/or cognitive deficits. Furthermore, our findings were obtained with a relatively small sample and therefore they should be considered as preliminary and replicated in further experiments. Finally, additional studies should be carried out to determine if variations in sLF levels predict AD progression.

## Conclusions

In summary, these findings provided first evidence relating sLF to regional Aβ load, cortical integrity, and poorer memory in clinically normal older adults. In particular, our results suggested that global Aβ load plays a major role in boosting associations between sLF and abnormal patterns of cortical thickness and cortical glucose metabolism, likely signaling early dysregulation of the immune system. Although these findings are preliminary, they represent a step further into the utilization of sLF as a non-invasive biomarker of cerebral vulnerability in the general aging population.

## Supplementary Information

**Additional file 1.**

## Data Availability

The datasets employed in the current study are available from the corresponding author on reasonable request.

## Abbreviations

Aβ: Amyloid-β
AD: Alzheimer’s disease
FDG: 2-[18F]fluoro-2-deoxy-D-glucose
FBB: Florbetaben
MCI: Mild cognitive impairment
MMSE: Mini Mental State Examination
MRI: Magnetic resonance imaging
PET: Positron emission tomography
PET-CT: Positron emission tomography-computed tomography
PVC: Partial volume correction
sLF: Salivary lactoferrin
SUVR: Standardized uptake value ratios
TMT: Trail making test
