# Supplementary material for: Salivary lactoferrin is associated with cortical amyloid-beta load, cortical integrity, and memory in aging
Source: Alzheimers Res Ther. 2021 Sep 6;13:150. doi: 10.1186/s13195-021-00891-8 (PMC8422723; doi:10.1186/s13195-021-00891-8)
Supplement: Supplementary file 1 — Additional file 1. [file 13195_2021_891_MOESM1_ESM.docx]

**Supplementary Material**

**Salivary lactoferrin is associated with cortical amyloid-beta load, cortical integrity, and memory in aging**

Lucia Reseco^1,2^, Mercedes Atienza^1,2^, Marina Fernandez-Alvarez^1,2^, Eva Carro^2,3^, Jose L. Cantero^1,2*^

**1. Effect of age and sex on cognition**

To evaluate the effect of age and sex on cognition, we first obtained a composite score for each cognitive domain (i.e., memory, working memory, attention, language, and executive function). As cognitive domain is a within-subjects factor, we applied mixed effects modeling, which allows intercepts to vary across participants. In particular, we specified a three-step mixed effects model. The first model only included the intercept, the second model included the main effects of cognitive domain, sex and age, and the third model included the interaction of cognitive domain with either sex or age. The model including the three main predictors (i.e., cognitive domain, sex and age) provided a better fit to the data than the intercept-only model ($\chi^{2}$ = 18.9, p = 0.004). The additive model showed a significant main effect of age on cognition (*F*_1,74_ = 20.3, p = 0.00002) but it did not reveal significant differences between sexes or between cognitive domains. However, the ANOVA applied to compare the additive and interaction models showed that the cognitive domain moderated the association between age and cognition ($\chi^{2}$ = 14.9, p = 0.005). Post hoc analyses indicated that the association of age with memory was more negative than the association of age with other cognitive domains. The cognitive domain × sex interaction was not significant.
